# Altered cross-talk between the hypothalamus and non-homeostatic regions linked to obesity and difficulty to lose weight

**DOI:** 10.1038/s41598-017-09874-y

**Published:** 2017-08-30

**Authors:** Oren Contreras-Rodríguez, Raquel Vilar-López, Zane B. Andrews, Juan F Navas, Carles Soriano-Mas, Antonio Verdejo-García

**Affiliations:** 1 Psychiatry Department, Bellvitge University Hospital, Bellvitge Biomedical Research Institute-IDIBELL, and Centro de Investigación Biomédica en Red de Salud Mental (CIBERSAM-17), Barcelona, Spain; 2 Red de Trastornos Adictivos, University of Granada, Granada, Spain; 3 Mind, Brain and Behavior Research Center, Universidad de Granada, Granada, Spain; 4 Monash Biomedicine Discovery Institute, Metabolic Disease and Obesity Program, Department of Physiology, Monash University, Melbourne, Australia; 5 Department of Experimental Psychology, University of Granada, Granada, Spain; 6 Department of Psychobiology and Methodology of Health Sciences, University Autònoma de Barcelona, Barcelona, Spain; 7 School of Psychological Sciences and Monash Institute of Cognitive and Clinical Neurosciences, Monash University, Melbourne, Australia

## Abstract

Interactions between the hypothalamus and non-homeostatic regions may contribute to explain the difficulty to lose weight in obesity, an assumption never tested in human longitudinal studies. We investigated whether the functional connectivity between the medial and lateral hypothalamus (MH and LH) and corticostriatal regions differs between individuals with excess weight (n = 42) and normal weight (n = 39) using a seed-based resting-state approach. In addition, we examined the longitudinal association between functional connectivity and weight loss in a 3-month follow-up diet. Results showed that participants with excess weight had increased connectivity between the MH and the striatum and subgenual anterior cingulate cortex, and decreased connectivity with the middle frontal gyrus, and the bed nucleus of the stria terminalis (BNST), as well as a decreased connectivity between the LH and the cerebellum. Decreased connectivity between the MH and the posterior part of the BNST, and between the LH and the cerebellar cortex, predicted a greater percentage of weight loss. Functional connectivity measures explained 36% of the 3-month weight change among individuals with excess weight. We conclude that altered functional connectivity between homeostatic-hypothalamic regions and non-homeostatic corticostriatal and cerebellar regions is linked to obesity and difficulty to lose weight.

## Introduction

Obesity is a major health problem in Western societies, as it is strongly associated with morbidity and mortality^1^. Current biomedical treatments for obesity do not work for most people, and treated individuals typically experience difficulties to maintain weight loss over extended periods of time^2, 3^. The role of brain systems for energy regulation on the development and maintenance of obesity has been long acknowledged. Classical studies in animals showed that localized lesions in distinct regions of the hypothalamus could lead to hyperphagia or suppression of spontaneous food intake^4^, leading to the definition of the lateral hypothalamus as the feeding center and the ventromedial hypothalamus as the satiety center. In addition to energy regulation mechanisms, in the current obesogenic environment food is also consumed due to its reward and hedonic aspects^5^. However, there has been relatively little research on the interaction between the hypothalamus and other brain regions involved in the coding of food-related reward and related choices.

Influential theories have proposed a link between the corticostriatal systems implicated in reward processing and decision-making and weight regulation^6, 7^. Neuroimaging studies have shown that people with excessive body mass index (BMI) have an altered response to food stimuli in reward processing regions (i.e., striatum, insula/ somatosensory cortex and amygdala)^8–13^. Furthermore, increased connectivity between some of these regions (i.e., striatum and somatosensory cortex) predict difficulty to lose weight^14^. Conversely, greater activity in frontal regions strongly involved in cognitive control and decision-making, such as the superior frontal gyrus, are linked to higher levels of dietary restrain^15^.

Homeostatic and reward-decision-making systems are meaningfully intertwined^16^. The neuropeptides and hormones that regulate homeostasis and energy balance in hypothalamic circuits also modulate neural activity in corticostriatal regions involved in reward processing^17, 18^. Furthermore, heightened functional connectivity between the medial hypothalamus and the ventral and medial prefrontal cortices, as well as striatal regions, has been reported in excess weight individuals during fasting^19, 20^. Thus, there is a need to better understand how homeostatic and non-homeostatic brain systems communicate, and how this communication relates to weight regulation^21^. To our knowledge, only one study to date has examined this association in obese patients during reduced weight maintenance with placebo or leptin injections^22^. However, their results were limited by the lack of a normal weight comparison group, and the lack of clinically meaningful longitudinal measures (i.e., weight loss).

This study aimed to assess differences in the connectivity between homeostatic (medial and lateral hypothalamic regions) and non-homeostatic brain regions among normal- versus excess-weight participants (=BMI ≥ 25, including people with overweight and obesity), and to use connectivity metrics to predict weight loss after dietary counselling in a 3-month follow-up. According to the previous literature, we hypothesized that excess compared to normal weight participants will show: *increased* functional connectivity between the medial hypothalamus and reward-related regions (i.e., striatum);^19, 20^ and *decreased* functional connectivity between the medial and lateral hypothalamus and cognitive control regions (i.e., superior and middle frontal gyri)^15, 23^. Moreover, functional connectivity in the above-described networks will predict weight change in excess relative to normal weight participants at 12-week follow-up^8–13, 23^.

## Results

### Sociodemographic variables

Participants with excess weight and normal weight did not significantly differ in age, educational levels or male/female ratios (Table 1).

**Table 1:** Demographics and clinical characteristics of the study groups. Except for sex, all values are mean ± SD. For % weight change, data of 27 excess weight is provided after discarding one outlier.

	Normal weight (n = 39)	Excess weight (n = 42)	p
Age (years)	33.07 ± 6.73	33.59 ± 6.16	0.72
Education (years)	18.18 ± 3.75	17.50 ± 3.77	0.42
Sex (men/women)	18(46.2%)/21(53.8%)	20(47.6%)/22(52.4%)	0.89
BMI baseline (kg/m2)	22.09 ± 1.74	30.51 ± 3.63	0.00
min/max	19/24.8	25.20/38.30	
Hunger before fMRI	15.03 ± 19.07	16.27 ± 18.72	0.77
Hunger after fMRI	39.59 ± 28.62	44.20 ± 25.45	0.46
	**Normal weight (n = 24)**	**Excess weight (n = 28)**	
Weight baseline (kg)	64.07 ± 8.57	89.69 ± 12.86	0.00
min/max	48.4/78.4	61.7/113.1	
Weight post-diet (kg)	63.72 ± 9.22	87.86 ± 12.76	0.00
min/max	49.1/80.2	65.1/113.6	
Weight change (kg)	0.49 ± 1.59	−1.55 ± 4.48	0.04
min/max	−3.5/6.3	−9.5/5.5	
Weight change (%)	0.84	−2.14	0.43
Participants losing weight (%)	41.7	64.8	0.10

### Medial and Lateral Hypothalamic functional connectivity

*Within-group* positive and negative functional connectivity maps of the MH and LH depicted highly distinct non-overlapping networks in both normal and excess weight participants. In brief, the MH positive connectivity map predominantly included ventral regions, whereas the LH positive connectivity map largely included frontal cortices. Negative connectivity maps for each seed highly overlap those brain regions positively associated with the other seed (i.e. brain regions showing a positive connectivity with MH, showed a negative connectivity with LH, and vice versa). See the Supplementary Results, Fig. S2 and Table S1 for further details about the specific brain regions included in the MH and LH functional connectivity maps.

#### Between-group differences

**Figure 1:**
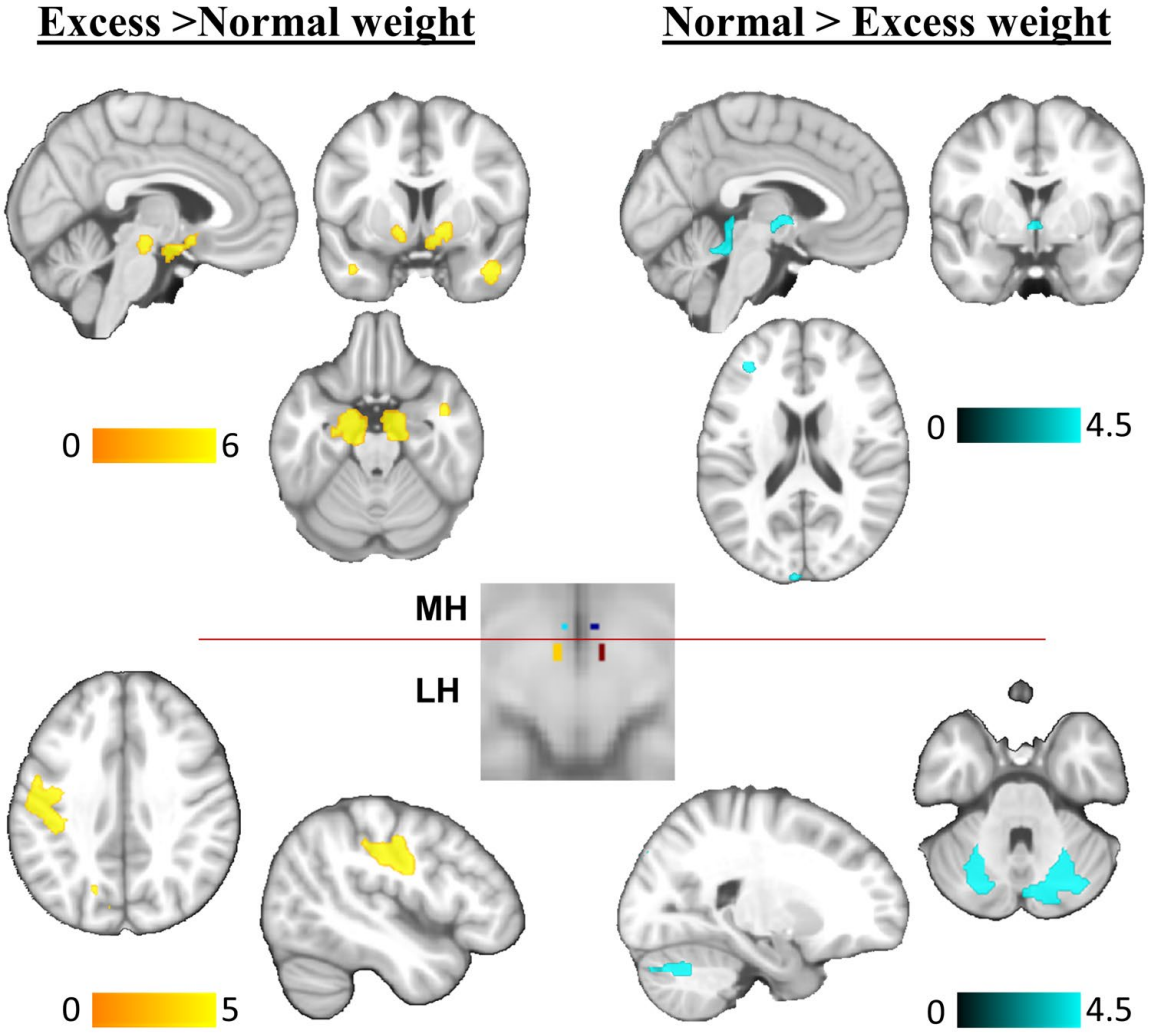
Between-group differences in the functional connectivity of the MH and the LH seeds. Brain regions showing increased (yellow) or decreased (blue) functional connectivity with the medial (**MH**) and lateral (**LH**) hypothalamus in excess weight compared to normal weight participants. The placement of the MH and LH seed regions of interest are presented in the square located in the middle of the figure. Seeds are presented in standard neuroanatomical space (Montreal Neurological Institute, MNI). The right hemisphere corresponds to the right side of axial and coronal views. The color bars indicate t-values.

*Medial Hypothalamus*:Excess weight participants compared to normal weight controls showed increased functional connectivity between the MH and a cluster that encompassed the subgenual anterior cingulate cortex, the ventral striatum, the substantia nigra, and parts of the temporal cortex. Moreover, excess weight participants showed decreased functional connectivity between the MH and the middle frontal gyrus, the bed nucleus of the stria terminalis, and the cerebellar vermis (lobule V of the vermis, extending to the adjacent brainstem colliculi) (Fig. 1, Table 2).

**Table 2:** Between group differences in the functional connectivity of the medial (MH) and lateral (LH) hypothalamic seeds.

Seed	Brain region	x, y, z	t	CS	Direction
**MH**					
	Subgenual Anterior Cingulate	6, 8, −14	3.4	1089*	Excess > Normal weight
	Ventral striatum	14, 4, −8	4.7	1089*	Excess > Normal weight
		−12, 4, −6	3.4	1089*	Excess > Normal weight
	Substantia nigra	10, −12, −22	5.9	1089*	Excess > Normal weight
		−10, −8, −18	5.0	1089*	Excess > Normal weight
	Temporal pole	44, 2, −30	3.8	158	Excess > Normal weight
		−36, 16, −36	4.4	32	Excess > Normal weight
	Middle frontal gyrus	−30, 46, 6	3.9	41	Normal > Excess weight
	Bed nucleus stria terminalis	−4, −4, −2	3.7	29	Normal > Excess weight
	Cerebellar vermis (lobule V)	2, −42, −20	4.4	160	Normal > Excess weight
**LH**					
	Somatosensory cortex	−50, −18, 30	4.8	487	Excess > Normal weight
	Cerebellum (lobules VI, Cr I)	−34, −62, −36	4.5	150	Normal > Excess weight

Coordinates (x, y, z) are given in Montreal Neurological Institute (MNI) Atlas space. *Indicates part of the large cluster. All results herein surpassed a height threshold of P < 0.001 and a cluster of 232 mm^3^ (29 voxels) for the MH, and 1072 mm^3^ (134 voxels) for the LH, explored inside the mask of within-group effects.

*Lateral hypothalamus*:Excess weight participants showed increased functional connectivity between the LH and the left primary somatosensory cortex. Moreover, excess weight participants showed decreased functional connectivity between the LH seed and a cerebellar cluster comprising lobules VI and Cr I (Fig. 1, Table 2).

All the above between-group functional connectivity differences in MH and LH networks remained significant when group effects were examined in a Repeated Measures General Linear Model in SPSS (intra-subject factor: extracted signal from right/left MH or LH seeds; inter-subject factor: normal and excess weight groups; sex and age were entered as covariates of no interest).

### Weight loss results

Participants with excess weight showed a significant reduction of weight in the 3-month follow-up (t = 2.92, p = 0.007). Specifically, 18 participants with excessive BMI lost weight, although 9 participants gained weight (min/max percentage of weight change −9.5/5.5, respectively). There were no significant weight changes among participants with normal weight (t = −1.52, p = 0.14), who showed expected mild fluctuations in weight status over the 3-month period^24^ (Table 1).

### Correlations between functional connectivity metrics and percentage of weight change

The within-group associations between the percentage of weight loss and the functional connectivity of the MH and the LH are detailed in the Supplementary Material (Supplementary Results, Fig. S3, and Table S2).

**Figure 2:**
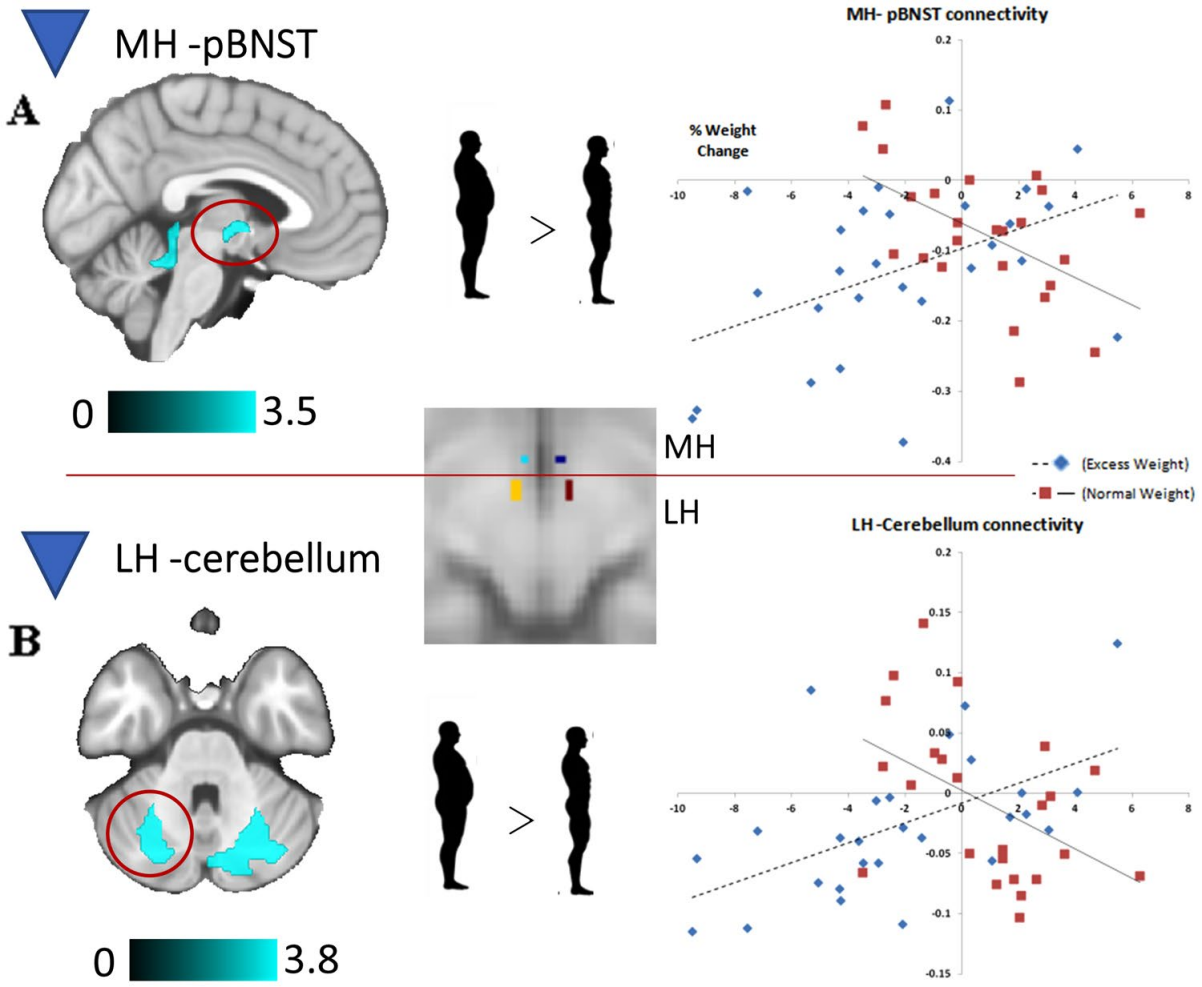
Brain regions displaying a significant between-group interaction in the relationship between MH (**A**) and LH (**B**) functional connectivity and percentage of weight change. The right hemisphere corresponds to the right side of axial view. The color bars indicate t-values. Scatter plots represent the correlations between the indicated clusters (top, posterior part of the bed nucleus of the stria terminalis [pBNST]; bottom, left cerebellum) and percentage of weight change (positive values indicate weight gain at follow-up, whereas negative values indicate weight loss).

In *between-group comparisons*, we found two significant interactions: one in the correlation between percentage of weight change and the functional connectivity of the MH seed with the posterior part of the bed nucleus of the stria terminalis (pBNST), and a second one in the correlation between percentage of weight change and the functional connectivity of the LH seed with the cerebellar cortex (VI lobule) (Fig. 2, Supplementary Table S2). In both cases, lower functional connectivity metrics correlated with a greater percentage of weight loss in excess weight individuals but not in normal weight participants. Additional results that did not map on the regions that differed between participants with excess weight and normal weight are reported in Supplementary Table S2.

To elucidate the overall contribution of MH-pBNST and LH-cerebellum functional connectivity to the percentage of weight change, we entered these connectivity estimates as independent predictors in a linear regression model, controlling for the sex and age of the participants. As expected, in excess weight participants, percentage weight change was positively predicted by the connectivity between MH and pBNST (B = 0.37, p = 0.044) and LH– cerebellum (B = 0.50, p = 0.004), while sex and age were not significant predictors (R^2^ = 0.137, p = 0.171). Overall, functional connectivity between these regions accounted for a 36% of the variance of percentage of weight change in excess weight participants over and above sex and age (F_Change 2,22_ = 7.735, p = 0.003, R^2^_Change_ = 0.356).

#### Post Hoc Analyses

To examine if the above significant group-interactions in the association between the percentage of weight change and the functional connectivity in the MH-BNST and the LH-cerebellum networks were driven by gaining or losing weight during diet, we conducted new separate full-factorial analyses (one for the MH, another for the LH), with a 2 × 2 design (Group [Normal weight vs Excess weight] x Weight Change [Gain or Loss]). This model showed that the relationship between the functional connectivity of the LH-cerebellum network (VI lobule) and the percentage of weight change was specific to the participants who lost weight. That is, lower functional connectivity in the LH-cerebellar network correlated with a greater percentage of weight loss in excess weight individuals who lost weight, whereas associations were not significant in normal weight participants counterparts. No significant effects were found for individuals who gained weight in this later network, and non-significant interactions were found for the MH-BNST network when participants who lost or gained weight were compared.

## Discussion

We observed that excess weight participants showed increased functional connectivity between the MH and the ventral striatum, subgenual anterior cingulate cortex and substantia nigra, as well as between the LH and the somatosensory cortex. Conversely, they have decreased connectivity between the MH and the BNST and the middle frontal gyrus. Connectivity between MH and LH and different cerebellar subregions was also decreased in excess weight participants. These findings were linked to clinically meaningful outcomes. Decreased functional connectivity between the MH and the pBNST and between the LH and the VI lobule of the cerebellum predicted greater weight loss at 3-months follow-up in excess weight individuals. Functional connectivity indices accounted for a 36% of weight change in excess weight individuals.

The finding of increased functional connectivity between the MH and the ventral striatum/subgenual anterior cingulate cortex in excess weight individuals concurs with our initial hypothesis and previous findings showing that resting-state functional connectivity in the hypothalamus is associated with greater reward valuation of food among excess weight participants^19^. We also found the hypothesized reduced connectivity with the middle frontal gyrus, in agreement with previous reports^15, 23^, although this finding was limited to the MH. Functional alterations in prefrontal cortices have been related to problems in self-regulation towards healthy eating^15, 25^. Overall, our findings align with the view that hyper-connectivity of reward regions and hypo-functioning of cognitive control regions may reduce the homeostatic control of feeding behavior^26^. However, the described functional connectivity alterations did not predict weight change in excess weight individuals as hypothesized based on previous similar studies^8–13, 15, 23^. Furthermore, also contrary to previous findings^19^, excess weight individuals showed increased functional connectivity between the LH and the left somatosensory cortex, overlapping with areas where the mouth, lips and tongue are represented, as well as gustatory areas^27^. This region has shown increased baseline metabolism in excess weight individuals, and it is suggested that this hyper-metabolism could enhance the salience of food stimuli via dopaminergic pathways^14, 28^. Thus, it is possible that LH-somatosensory cortex hyper-connectivity contributes to obesity by fostering the appeal of food.

In excess weight participants, lower functional connectivity between the MH with the pBNST, which was indeed decreased in comparison with normal weight controls, was associated with weight loss. The pBNST facilitates behavioral adaptation to stress^29, 30^ via inhibition of HPA activity^31^. Specifically, GABAergic neurons in the pBNST convey inhibitory control signals from the medial prefrontal cortex to the parvocellular paraventricular nucleus of the MH^32, 33^. Dieting in humans have been associated with increases in emotional stress^34^ and cortisol levels^35, 36^, and rodent studies have shown that previous restriction experiences produce changes in BNST neurochemistry that leads to an increase in stress sensitivity and over-consumption of high-fat food^37, 38^. These alterations may contribute to a reduction in the inhibitory projections from the pBNST GABAergic neurons to the paraventricular hypothalamus, therefore linking caloric restriction with HPA hypersensitivity^31, 39^. Accordingly, the excess weight individuals that showed greater anticorrelation in the MH-pBNST connection – purportedly, the ones with higher inhibition on the HPA axis– were found to be more prone to lose weight, whereas those with higher positive connectivity –reflecting HPA axis sensitization– were found to lose less weight. Future studies are needed to formally evaluate this mechanism and its direct impact on weight change, as modulation of presynaptic BNST-GABAergic inputs to the LH has been associated with feeding regulation in mice^40^.

Likewise, decreased functional connectivity between the LH and the cerebellum was also associated with weight loss in excess weight individuals, and post-hoc analyses showed that this finding was specific of participants who lost weight over the 3-months period. Most preclinical and human research has shown that cerebello-hypothalamic projections have a key role in somatic-visceral integration -i.e., glucemia/glucose, gastric motility- and thus in feeding regulation^41^. In normal weight individuals, higher baseline cerebellar activation has been recently associated with higher levels of dietary restraint^42^, whereas decreased cerebellar activity during a decision-making task has been associated with weight gain^43^. Indeed, higher metabolism in the cerebellum has been found in a sample of obese individuals who lost an average of 11.6% in BMI after 1–2 years of Gastric Stimulator (GS) implantation^44^, thought to increase the brain sensitivity to gastric signals^45^. Moreover, a study that evaluated the connectivity of the hypothalamus before and after weight loss, via Roux-in-Y gastric bypass, showed higher connectivity (i.e., decreased anticorrelation) between the hypothalamus and the cerebellum in obese individuals who lost weight^46^. However, our results showing that dysconnectivity between the cerebellum and the hypothalamus is associated with weight loss may suggest that weight changes after a dietary intervention may not be driven by somatic visceral signs. In this context, Wang and colleagues^44^ suggested that weight loss associated with GS stimulation in excess weight individuals might be possible due to concomitant reductions in the limbic brain responses to food or food-conditioned stimuli. Our map of positive correlations with weight change in excess weight individuals support these suggestions, as we show that, together with the cerebellum, weight loss was also associated with a reduced connectivity between the LH and regions commonly related to external eating -i.e. extended amygdala, anterior insula (ref. 47, see ref. 48 for implications of the LH-amygdala network in external eating). This is also congruent with the cerebellar role in conditioning and emotional processing^49^ and with studies showing cerebellar hyperactivation to food cues in excess weight samples^50^.

This study has important strengths, such as the adequately powered sample size, the control group and the prospective design. We identified functional connections that robustly predict individual differences in weight loss (36% of the variance). However, one limitation is that prospective analyses were conducted in a subgroup representing only 67% of the original sample. Nonetheless, this attrition rate is consistent with that reported in meta-analytic research on obesity interventions^51^, and participants in the follow-up subgroups did not significantly differ from the main sample in antecedent variables. An additional limitation refers to the lack of a concurrent index of stress to substantiate the assumption that weight loss associates with the ability to regulate diet-induced stress through changes in the connectivity within the MH-BNST network. Finally, our results cannot speak to the potential normalization of functional connectivity after weight loss. Future studies using longitudinal neuroimaging designs are needed to address this important clinical question.

We conclude that the connectivity between homeostatic-hypothalamic and non-homeostatic brain regions is relevant to explain individual differences in weight loss among people with overweight and obesity. This knowledge could be applied to develop prognostic signatures of weight loss and surrogate markers of treatment response. They can also inform novel neuromodulation approaches for the treatment of obesity.

## Methods

### Participants

Forty-two participants with excess weight (BMI > 25) and 39 with normal weight (BMI 18 to 24.9) participated in the study. This sample overlaps with that of a previous study, where we analyzed the functional connectivity of the brain reward system^14^. The sample size was determined by a power analyses based on a medium effect size for the comparison between normal and excess weight participants, an estimated power of 0.8, and an alpha level of 0.05. Participants were recruited via general hospitals and community advertisement (i.e., local press, radio and social media), and enrolled if they were aged 18 to 45, right-handed, and had BMIs > 18 (see a Participant flow diagram in the Supplementary Fig. S1). Exclusion criteria were: (i) history of brain injury or severe medical conditions affecting the central nervous system, (ii) history of substance use, major depression or psychosis, (iii) self-reported use of psychotropic medication, and (iv) morbid obesity (BMI ≥ 40). The Human Research Ethics Committee of the University of Granada approved the study according to the Declaration of Helsinki, and all participants provided informed consent.

Participants conducted two assessment sessions. At baseline they (i) were weighed and inclusion criteria confirmed; (ii) underwent a functional Magnetic Resonance Imaging (fMRI) scanner; and (iii) had a 30-minute appointment with a professional dietitian, who provided personalized weight loss strategies to the participants with excess weight, and standard dietary guidelines for healthy eating to the participants with normal weight. At 12-week follow-up, excess weight (n = 28, 66% of the original sample) and normal weight (n = 24, 62% of the original sample) participants were re-assessed to calculate weight change relative to baseline. Twelve weeks is the standard benchmark to assess the outcomes of weight loss interventions^52^.

### Measures

#### Imaging data Acquisition and preprocessing

All participants were scanned at the same time of the day (between 4:00 and 6:00 PM), timed after the usual lunch break in Southern Europe (2:00–4:00 PM) to standardize homeostatic status across participants. Pre-scanner ratings of hunger (0–100 VAS) did not differ between groups at baseline and follow-up (Table 1). Participants underwent a 6-minute resting-state scan. They were instructed to lie still with eyes closed. We used a 3.0 Tesla clinical MRI scanner, equipped with an eight-channel phased-array head coil (Intera Achieva Philips Medical Systems, Eindhoven, The Netherlands). A T2*-weighted echo-planar imaging (EPI) was obtained (TR = 2000 ms, TE = 35 ms, FOV = 230 × 230 mm, 96 × 96 pixel matrix; flip angle = 90°, 21 4-mm axial slices, 1-mm gap, 180 whole-brain volumes). The sequence included four initial dummy volumes to allow the magnetization to reach equilibrium.

#### Percentage weight change

Percentage weight change was calculated by dividing weight change (subtraction of baseline weight from follow-up weight) by the baseline weight, and multiplying the resulting value by 100 [(weight change/baseline weight) * 100]. This change index is clinically relevant as it reflects individual differences in weight loss accounting for baseline weight. Greater positive values reflected higher percentage of weight gain, whereas greater negative values reflected higher percentage of weight loss. One outlier in the excess weight participants (>2 SD from the mean) was excluded from the analyses using this measure, and hence the longitudinal sample comprised 27 excess weight and 24 normal weight participants.

### Image Preprocessing

The functional imaging data was processed and analyzed using MATLAB version R2008b (The MathWorks Inc, Natick, Mass) and Statistical Parametric software (SPM8; The Welcome Department of Imaging Neuroscience, London). Preprocessing steps involved motion correction, spatial normalization and smoothing using a Gaussian filter (FWHM 8 mm). Data were normalized to the standard SPM-EPI template and resliced to a 2 mm isotropic resolution in Montreal Neurological Institute (MNI) space. All images were inspected for potential acquisition and normalization artifacts. No participants were excluded because of artifacts or head displacements (>2 mm for translations and >2° for rotations in any x, y or z axis). Additionally, we compared both study groups for potential differences in movement for translations and rotations and found no significant differences [Mean Total Movement (SD), normal weight controls = 0.31 (0.18), excess weight participants = 0.31 (0.29), p = 0.34].

### Analyses

#### Behavioral

Sociodemographic and weight data, reported in Table 1, were analyzed using SPSS v. 20.0. Paired sample t-tests were used to compare the mean difference of all variables between normal and excess weight groups, as variables showed a normal distribution as assessed by Kolmogorov-Smirnov tests. As an exemption, chi-square tests were used to compare the sex, and the percentage of weight change and participants losing weight between the study groups.

#### Hypothalamic seed-based functional connectivity analyses

Medial and lateral hypothalamic subregions were identified in each hemisphere. Seeds of interest were placed in the medial hypothalamus (MH, x = +4, y = −2, z = −12) and the lateral hypothalamus (LH, x = +6, y = −10, z = −10) using 2-mm-radius spheres, according to validated methods^19, 53^. The MH seed included the arcuate nucleus, as well as the ventromedial and parts of the dorsomedial hypothalamus. The central voxel of the LH seed was located in the most posterior part of the region to minimize overlap with the MH seed and obtain maximally specific functional connectivity maps. Importantly, these seeds were spatially separated by more than 8 mm (>1 FWHM).

First-level (single subject) t-test maps were estimated for each MH and LH seed region by including its mean activity time courses (extracted using the Marsbar toolbox)^54^ together with nuisance signals as predictors of interest and no interest in whole-brain SPM8 linear regression analyses. Nuisance signals included the six head-motion parameters (3 translations and 3 rotations) and three time courses representing mean signal fluctuations in white matter, cerebrospinal fluid and the entire brain. Separate first-level analyses were carried out for right and left hemisphere seeds. A high-pass filter (128-seconds) was used to remove low-frequency drifts. Low-pass filtering was not used in agreement with recent guidelines for resting-state analyses^55^. Contrast images were generated for each participant by estimating the regression coefficient between each seed’s time series and all brain voxels and were then included in separate second-level full factorial models to assess for between-group effects.

#### Associations with percentage of weight change

We conducted whole-brain correlations in SPM8 between the functional connectivity maps of the MH and LH and percentage of weight change using second-level two-sample t-test models. These analyses included a single contrast image representing the bilateral connectivity of the MH and the LH seeds -created in the first-level (single-subject) models-, and percentage of weight change, in interaction with group, as a covariate of interest.

### Thresholding criteria

Significance threshold for all imaging analyses was determined by 1000 Monte Carlo simulations using AlphaSim as implemented in the SPM REST (Resting-State fMRI Data Analysis Toolkit) toolbox^56^. The input parameters to AlphaSim included an individual voxel threshold probability of p < 0.001, a cluster connection radius of 5 mm, and the actual smoothness of imaging data after model estimation. To correct for the within-group functional connectivity maps we selected the cluster size that satisfy a family-wise error rate correction of pFWE < 0.0125 to account for the multiple correlations performed (MH and LH, at right and left hemispheres in the first-level models). For the following post-hoc analyses a cluster size satisfying a family-wise error rate correction of pFWE < 0.05 was selected, as they were based on the within-group masks already corrected for multiple testing. Since the minimum cluster size varied across the different analyses, this value is specified in the corresponding Table of the results section. For illustrative purposes (i.e., in Figures) results are presented at a p < 0.005 threshold, unless indicated.

## Electronic supplementary material

Supplementary Information
