# Supplementary material for: Altered cross-talk between the hypothalamus and non-homeostatic regions linked to obesity and difficulty to lose weight
Source: Sci Rep. 2017 Aug 30;7:9951. doi: 10.1038/s41598-017-09874-y (PMC5577266; doi:10.1038/s41598-017-09874-y)
Supplement: Supplementary file 1 — Supplementary Information [file 41598_2017_9874_MOESM1_ESM.pdf]

Supplementary Information:

Altered cross-talk between the hypothalamus and non-homeostatic regions linked to obesity and difficulty to lose weight

Oren Contreras-Rodríguez, PhD, Raquel Vilar-López, PhD, Zane B. Andrews, PhD, Juan F Navas, MSc, Carles Soriano-Mas, PhD*, Antonio Verdejo-García, PhD

Supplementary Results

Supplementary Fig. S1. Participants flow-diagram

Supplementary Fig. S2. Within-group functional connectivity maps of the MH and LH. Positive (top panel) and negative (bottom panel) functional connectivity maps of the medial (positive: red; negative: green) and lateral (positive: yellow; negative: blue) hypothalamic seeds depicting highly distinct non-overlapping brain circuits between seeds in both normal and excess weight participants. The color bar indicates t-values. Results are displayed at the established threshold of $p<0.001$, uncorrected. The right hemisphere corresponds to the right side of axial and coronal views.

Supplementary Fig. S3. Within-group associations between the percentage of weight loss and the functional connectivity of the MH and the LH. Medial (MH, top panel) and lateral (LH, bottom panel) hypothalamic functional connectivity maps showing significant positive (+) and negative (-) associations with % weight change in normal and excess weight subjects. The right hemisphere corresponds to the right side of axial and coronal views. The color bar indicates t-values.

Supplementary Table S1. Within-group positive and negative functional connectivity maps for the medial (MH) and the lateral (LH) hypothalamic seeds. Coordinates (x, y, z) are given in Montreal Neurological Institute (MNI) Atlas space. Ns, indicates non-significant clusters. All results herein surpassed a height threshold of $p<0.001$ and 992 mm^3 (124 voxels).

Supplementary Table S2. **Correlations between the functional connectivity of the medial (MH) and lateral (LH) hypothalamic seeds and the percentage of weight change for normal and excess weight and participants and between-group interactions.** Coordinates (x, y, z) are given in Montreal Neurological Institute (MNI) Atlas space. Signs (+) and (-) refer to the direction of the significant correlations. Text in italics refers to the between-group connectivity correlates that map on the between-group differences. All results surpassed a height threshold of p<0.001. For within-group correlation effects a cluster of 232 mm3 (29 voxels) for the MH, and 1072 mm3 (134 voxels) for the LH was used, explored inside the mask of within-group effects. For the between-group correlation effects, a cluster of 80 mm3 (10 voxels) was used both for the MH and the LH, explored inside the mask of between-group effects for each seed, respectively.

Supplementary Results

Verification of the Medial and Lateral Hypothalamic Functional Connectivity Maps

The MH positive connectivity map predominantly included ventral regions such as the operculum orbitalis and the amygdalae bilaterally, and a cluster comprising the hippocampi that extended to the dorsal superficial thalamic nuclei, the superior temporal gryus and the fusiform gyrus. Distinctly, the LH positive connectivity map largely included frontal cortices such as the dorsomedial prefrontal cortex, rostral sections of the anterior cingulate cortex and the inferior frontal gyri extending to the operculum opercularis, and a cluster comprising the bed nucleus of the stria terminalis, striatal nuclei, and anterior and middle portions of the insulae (see also Fig. S1).

The negative functional connectivity maps of the MH and LH were also highly distinct and they highly contained those brain regions positively associated with the other seed (i.e. brain regions showing a positive connectivity with MH, showed a negative connectivity with LH, and viceversa). In that way, the MH negative connectivity map predominantly included frontal cortices (e.g. middle and inferior frontal gyri, rostral anterior cingulate cortex, operculum opercularis), striatal regions and the bed nucleus of the stria terminalis, whereas the LH negative connectivity map included the amygdala-hippocampus complex, and superior temporal and fusiform gyri (see also Fig. S1).

Within-group correlations with percentage of weight change

Normal weight: In normal weight participants, weight loss was associated with an increased functional connectivity between the MH seed and the right posterolateral orbitofrontal cortex, and with a decreased connectivity between the LH and the posterior cingulate cortex (see also Table S2).

Excess weight: In excess weight participants, weight loss was associated with a decreased functional connectivity between the MH seed and the BNST, as well as with a decreased connectivity between the LH seed and the left amygdala-hippocampus complex, and the right anterior insula (see also Table S2). Additionally, in these participants weight loss was associated with an increased connectivity between the MH seed and the right amygdala.

Multiple Regression Analyses

Data met all assumptions required for multiple regression analysis. An analysis of standard residuals was carried out, which showed that the data contained no outliers (std. residuals min = -1.75, std. residuals max = 1.70). Tests to see if the data met the assumption of collinearity indicated that multicollinearity was not a concern (MH-pBNST, tolerance = 0.99, VIF = 1.002; LH-cerebellum, tolerance = 0.99, VIF = 1.002). The data met the assumption of independent errors (Durbin -Watson value= 1.61). The histograms of standardized residuals indicated that the data contained approximately normally distributed errors, as did the normal P-P plots of standardized residuals, which showed points that were not completely on the line, but close. The scatterplot of standardized predicted values showed that the data met the assumptions of homogeneity of variance and linearity. Finally, the data also met the assumptions of non-zero variances (% weight change, variance = 15.369; MH- pBNST, variance = 0.923; LH-cerebellum, variance = 0.923).

Supplementary Fig. S1. Participants flow-diagram.

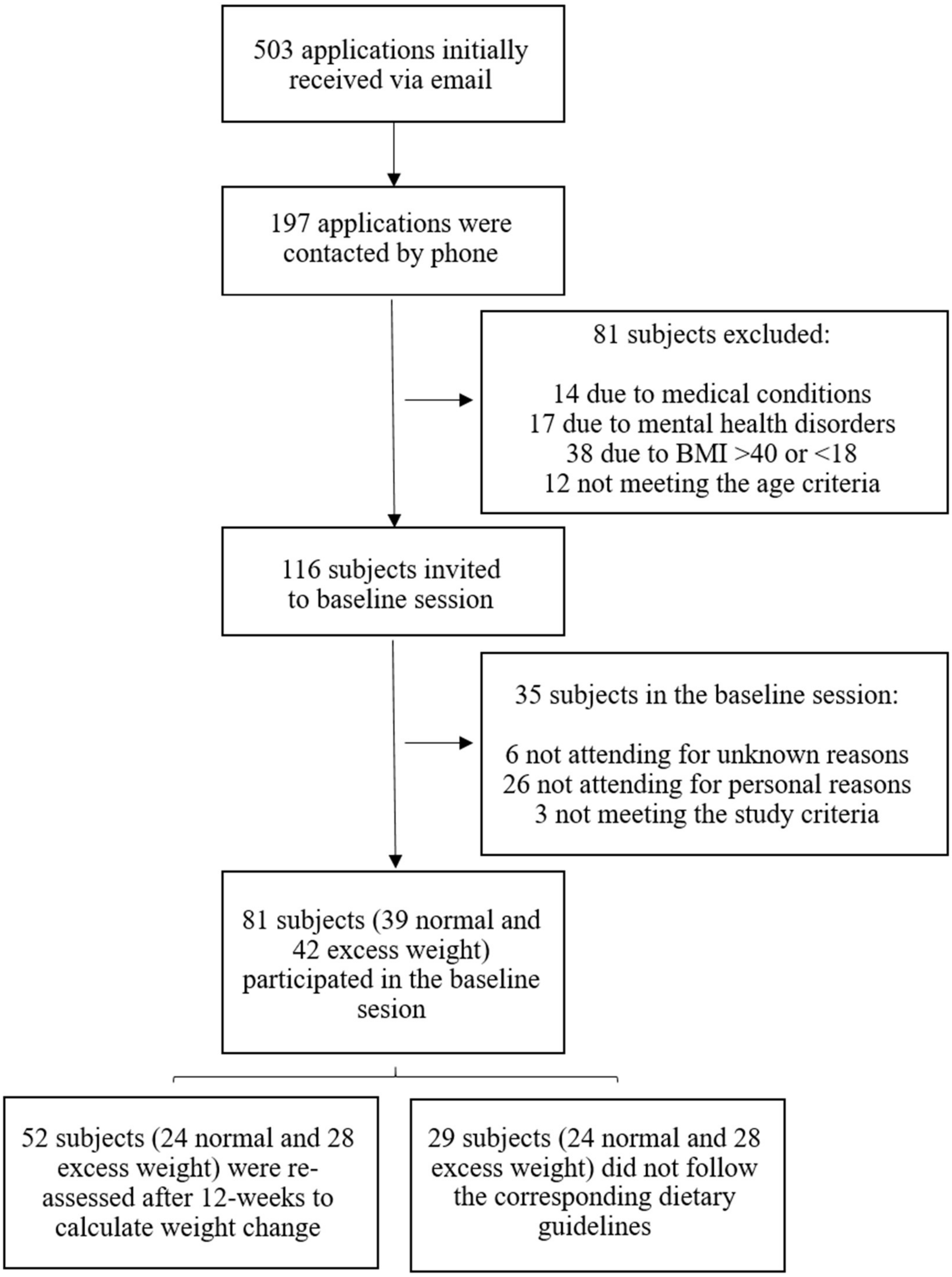

Supplementary Fig. S2. Within-group functional connectivity maps of the MH and LH. Positive (A) and negative (B) functional connectivity maps of the medial (positive: red; negative: green) and lateral (positive: yellow; negative: blue) hypothalamic seeds depicting highly distinct non-overlapping brain circuits between seeds in both normal and excess weight participants. The color bar indicates t-values. Results are displayed at the established threshold of $p<0.001$, uncorrected. The right hemisphere corresponds to the right side of axial and coronal views.

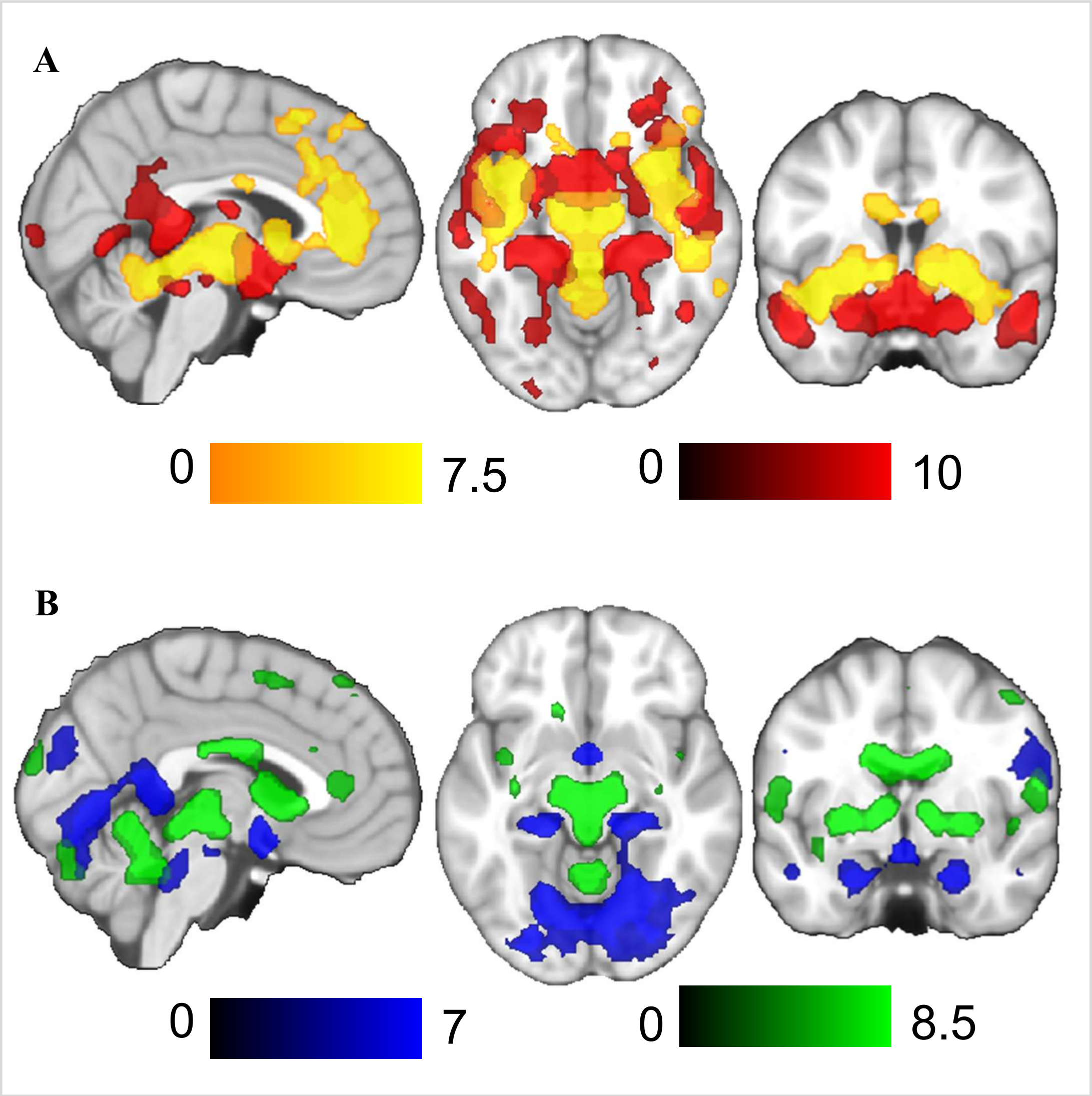

Supplementary Fig. S3. Within-group associations between the percentage of weight loss and the functional connectivity of the MH and the LH. Medial (A) and lateral (B) hypothalamic functional connectivity maps showing significant positive (+) and negative (-) associations with % weight change in normal and excess weight subjects. The right hemisphere corresponds to the right side of axial and coronal views. The color bar indicates t-values.

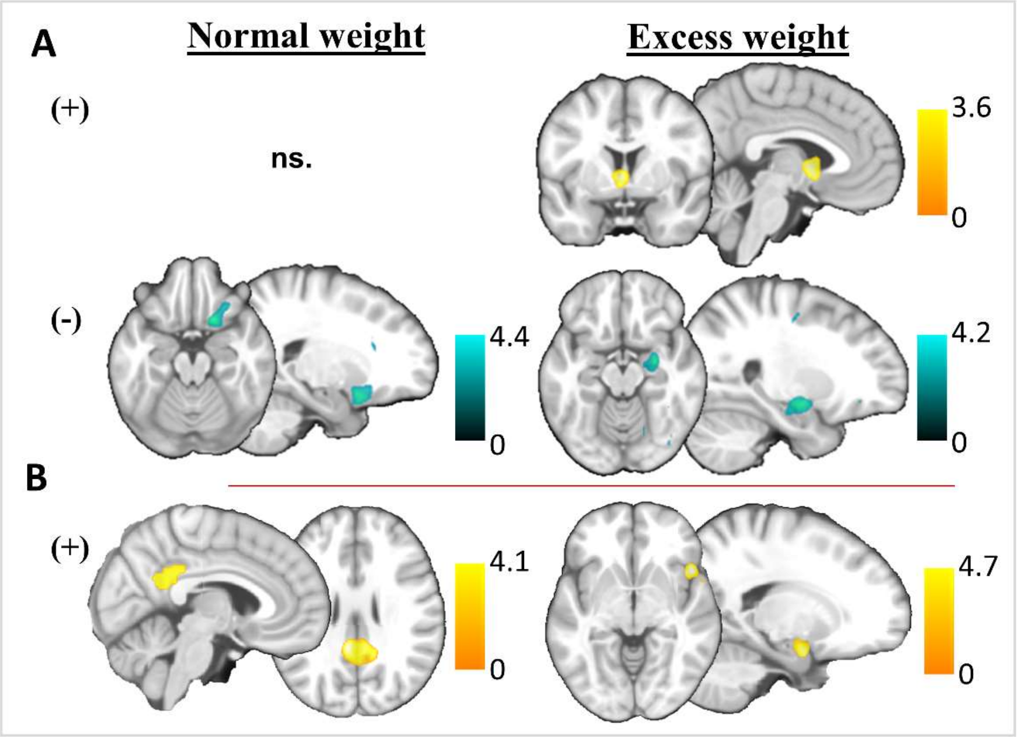

Supplementary Table S1. Within-group positive and negative functional connectivity maps for the medial (MH) and the lateral (LH) hypothalamic seeds. Coordinates (x, y, z) are given in Montreal Neurological Institute (MNI) Atlas space. Ns, indicates non-significant clusters. All results herein surpassed a height threshold of p<0.001 and 992 mm^3 (124 voxels).

Seed	Connected region	Normal weight		Excess weight	
		**x, y, z**	**t**	**x,y,z**	**t**
**MH**		*Positive connectivity*			
	Operculum orbitalis	36, 24, -16	8.1	32, 28, -20	5.5
		-36, 22, -18	7.6	-28, 26, -20	5.7
	Amygdala	24, 2, -20	6.5	24, 0, -18	5.4
		-26, 2, -18	8.4	-26, 8, -18	5.5
	Hippocampus	22, -32, -10	10.0	26, -28, -14	8.2
		-28, -30, -14	8.9	-32, -24, -18	6.2
	Superior temporal cortex	54, -8, -20	7.8	54, -8, -18	6.8
		-52, -2, -18	8.1	-60, -14, -16	5.3
	Fusiform gyrus	46, -44, -20	4.4	48, -46, -18	5.5
		-46, -44, -20	5.2	-44, -46, -18	4.2
	Thalamus (dorsal superficial)	10, -18, 16	4.7	0, -10, 12	5.3
	Posterior cingulate		ns	6, -42, 32	3.3
		Negative connectivity			
	Inferior/middle frontal gyrus	42, 40, 10	5.1	48, 32, 14	4.8
			ns	-44, 38, 12	5.3
	Rostral anterior cingulate	12, 40, 14	4.4	-4, 36, 8	4.8
	Operculum opercularis	54, 16, 14	4.4	56, 8, 10	6.5
			ns	-56, 8, 8	5.9
	Precentral gyrus		ns	44, -8, 56	4.7
	Supplementary motor area		ns	0, 2, 58	4.4
	Bed Nucleus Stria Terminalis	-4, 8, 0	6.1	4, 8, 2	8.1
	Dorsal Caudate		ns	18, 18, 14	4.4
	Putamen	24, 2, -4	7.2	26, 0, -2	4.6
		-24, 0, -2	5.9	-26, -2, -2	5.8
	Supramarginal gyri	56, -22, 18	4.6	48, -18, 12	4.5
		-42, -30, 18	4.4	-58, -32, 14	5.4
	Occipito-Temporal cortex	-54, -66, 12	3.9	66, -34, 6	4.9
	Pons	12, -18, -26	5.4		ns
	Cerebellum (lobule Cr I)	-36, -70, -32	5.0	0, -50, -18	8.5
**LH**		*Positive connectivity*			
	Dorsomedial prefrontal cortex	8, 34, 48	4.0	6, 30, 32	5.4
	Rostral anterior cingulate	4, 42, 16	5.8	-4, 42, 8	4.7
	Operculum opercularis/Inferior frontal gyrus	42, 40, 4	5.1		ns
		-40, 34, -6	4.2		ns
	Supplementary motor area		ns	0, 12, 52	4.0
	BNST-Striatum-Insula	16, 14, 0	6.6	10, 8, 4	5.4
		-10, 8, -2	7.3	-6, 6, 4	4.1
	Middle temporal cortex	46, -32, -10	6.0	-46, -38, -8	4.5
	Cerebellum (lobule IV)	0, -48, -18	5.9	-8, -48, -12	4.4

	Negative connectivity			
Amygdala	24, 2, -22	6.7	22, -4, -26	4.6
	-24, 0, -22	6.9	-24, -8, -24	4.9
Hippocampus	20, -30, -14	6.5	24, -26, -22	6.2
	-20, -28, -20	5.9	-20, -30, -12	6.1
Superior temporal cortex	48, 10, -26	5.3		ns
	-48, 8, -24	4.4		ns
Fusiform gyrus	22, -64, -10	5.0	36, -62, -14	4.9
	-32, -64, -22	4.9	-32, -66, -20	5.1
Posterior cingulate	0, -48, 8	5.1	0, -48, 10	4.5
Somatosensory cortex	52, -12, 38	4.8		ns
	-54, -14, 36	5.9		ns
Occipital cortex	-8, -88, 26	6.4	-16, -96, 26	4.6
Brainstem		ns	2, -34, -26	6.3
Cerebellum (lobules VI, Cr I)		ns	30, -70, -30	6.5
		ns	-36, -60, -36	5.6

Supplementary Table S2. **Correlations between the functional connectivity of the medial (MH) and lateral (LH) hypothalamic seeds and the percentage of weight change for normal and excess weight and participants and between-group interactions.** Coordinates (x, y, z) are given in Montreal Neurological Institute (MNI) Atlas space. Signs (+) and (-) refer to the direction of the significant correlations. Text in italics refers to the between-group connectivity correlates that map on the between-group differences. All results surpassed a height threshold of p<0.001. For within-group correlation effects a cluster of 232 mm3 (29 voxels) for the MH, and 1072 mm3 (134 voxels) for the LH was used, explored inside the mask of within-group effects. For the between-group correlation effects, a cluster of 80 mm3 (10 voxels) was used both for the MH and the LH, explored inside the mask of between-group effects for each seed, respectively. Abbreviations: OFC, orbitofrontal cortex; HPC, hippocampus.

	Brain region		x, y, z	t	CS	Brain region		x, y, z	t	CS
	**Normal weight**					**Excess weight**				
**MH**										
						Bed nucleus	(+)	-2, 4, 2	3.6	30
	Posterolateral OFC	(-)	18, 14, -18	4.4	35	Amygdala	(-)	28, -8, -14	4.2	53
	**Normal weight > Excess weight**					**Excess weight > Normal weight**				
				ns		*Bed nucleus*		*-6, 6, -2*	*3.5*	*19*
	**Normal weight**					**Excess weight**				
**LH**										
	Posterior Cingulate	(+)	14, -48, 24	4.1	134	Amygdala-HPC	(+)	-14, 0, -22	4.5	143
						Insula	(+)	48, 16, -10	4.7	158
	**Normal weight > Excess weight**					**Excess weight > Normal weight**				
				ns		*Cerebellum*		*-14, -66, -20*	*3.8*	*23*
